# Optimal targeting of BCL-family proteins in head and neck squamous cell carcinoma requires inhibition of both BCL-xL and MCL-1

**DOI:** 10.18632/oncotarget.26563

**Published:** 2019-01-11

**Authors:** Thomas J. Ow, Cory D. Fulcher, Carlos Thomas, Pilib Ó Broin, Andrea López, Denis E. Reyna, Richard V. Smith, Catherine Sarta, Michael B. Prystowsky, Nicolas F. Schlecht, Bradley A. Schiff, Gregory Rosenblatt, Thomas J. Belbin, Thomas M. Harris, Geoffrey C. Childs, Nicole Kawachi, Chandan Guha, Evripidis Gavathiotis

**Affiliations:** ^1^ Department of Otorhinolaryngology-Head and Neck Surgery, Montefiore Medical Center/Albert Einstein College of Medicine, Bronx, NY, USA; ^2^ Department of Pathology, Montefiore Medical Center/Albert Einstein College of Medicine, Bronx, NY, USA; ^3^ School of Mathematics, Statistics, and Applied Mathematics, National University of Ireland Galway, Galway, Ireland; ^4^ Department of Biochemistry, Albert Einstein College of Medicine, Bronx, NY, USA; ^5^ Department of Surgery, Montefiore Medical Center/Albert Einstein College of Medicine, Bronx, NY, USA; ^6^ Department of Epidemiology & Population Health, Montefiore Medical Center/Albert Einstein College of Medicine, Bronx, NY, USA; ^7^ Department of Medicine (Oncology), Montefiore Medical Center/Albert Einstein College of Medicine, Bronx, NY, USA; ^8^ Department of Cancer Prevention & Control, Roswell Park Comprehensive Cancer Center, Buffalo, NY, USA; ^9^ Discipline of Oncology, Memorial University of Newfoundland, St. John's, NL, Canada; ^10^ Department of Radiation Oncology, Montefiore Medical Center/Albert Einstein College of Medicine, Bronx, NY, USA; ^11^ Department of Medicine (Cardiology), Montefiore Medical Center/Albert Einstein College of Medicine, Bronx, NY, USA; ^12^ Albert Einstein Cancer Center, Bronx, NY, USA

**Keywords:** head and neck squamous carcinoma, MCL-1, BCL-xL, navitoclax, A-1210477

## Abstract

Mechanisms of treatment resistance in head and neck squamous cell carcinoma (HNSCC) are not well characterized. In this study, HNSCC tumors from a cohort of prospectively enrolled subjects on an ongoing tissue banking study were divided into those that persisted or recurred locoregionally (n=23) and those that responded without recurrence (n=35). Gene expression was evaluated using llumina HumanHT-12-v3 Expression BeadChip microarrays. Sparse Partial Least Squares – Discriminant Analysis (sPLS-DA) identified 135 genes discriminating treatment-resistant from treatment-sensitive tumors. BCL-xL was identified among 23% of canonical pathways derived from this set of genes using Ingenuity Pathway analysis. The BCL-xL protein was expressed in 8 HNSCC cell lines examined. Cells were treated with the BCL-xL inhibitor, ABT-263 (navitoclax): the average half maximal inhibitory concentration (IC50) was 8.9μM (range 6.6μM – 13.9μM). Combining ABT-263 did not significantly increase responses to 2 Gy radiation or cisplatin in the majority of cell lines. MCL-1, a potential mediator of resistance to ABT-263, was expressed in all cell lines and HNSCC patient tumors, in addition to BCL-xL. Treatment with the MCL-1 inhibitor, A-1210477, in HNSCC cell lines showed an average IC50 of 10.7μM (range, 8.8μM to 12.7μM). Adding A-1210477 to ABT-263 (navitoclax) treatment resulted in an average 7-fold reduction in the required lethal dose of ABT-263 (navitoclax) when measured across all 8 cell lines. Synergistic activity was confirmed in PCI15B, Detroit 562, MDA686LN, and HN30 based on Bliss Independence analysis. This study demonstrates that targeting both BCL-xL and MCL-1 is required to optimally inhibit BCL-family pro-survival molecules in HNSCC, and co-inhibition is synergistic in HNSCC cancer cells.

## INTRODUCTION

Head and neck squamous cell carcinoma (HNSCC) is diagnosed in approximately 55,000 patients in the United States each year [1], and it is among the most common cancers worldwide [2]. HNSCC arises at sites in the upper aerodigestive tract, including the oral cavity, larynx, and pharynx. Treatment recommendations for patients with HNSCC are selected based on disease site, clinical stage, and the morbidity associated with different treatment options. Approximately 50% of patients with HNSCC present with American Joint Committee on Cancer (AJCC) stage III or IV disease [3–5], and the vast majority of these patients receive radiation or combination cisplatin-radiation as either first-line or adjuvant therapy [6, 7]. For the last two decades there has been a consistent locoregional failure rate of 20-30% that has shown little if any improvement over that time [6, 8]. Thus, one can surmise that many locoregional recurrences of HNSCC represent either re-growth of tumor cells that have survived cisplatin and/or radiation treatment or new malignant cells that have arisen within the treated field. To date, little is known about the molecular factors that contribute most significantly to treatment failure in HNSCC.

Evasion of apoptosis is a ‘hallmark of cancer’ [9]. Oncogenic stressors (eg. DNA damage, unchecked cell proliferation) are known to elicit the activation of apoptosis signaling, which serves as a defense against the process of tumorigenesis. Additionally, radiation and cytotoxic chemotherapy cause preferential tumor cell death via accumulation of fatal DNA damage, which can lead to apoptosis as one potential mechanism of tumor cell death. Thus, disruption of apoptosis signaling plays a key role in the development of cancer, cancer cell survival, and resistance to therapy.

The process of apoptosis is controlled by a balance among several initiators, mediators, and inhibitors. The BCL-2 family proteins work in concert to regulate the initiation of the intrinsic apoptosis pathway. Pro-survival BCL-2 proteins, such as BCL-2, BCL-xL, and MCL-1, inhibit BCL-2 pro-apoptotic effectors, such as BAX and BAK. The pro-apoptotic effectors promote apoptosis by forming pore complexes in the mitochondrial membrane leading to release of oxygen free-radicals and cytochrome c, which in turn activate the caspase cascade leading to programmed cell death [10]. Upregulation of pro-survival BCL-2 proteins is a known mechanism by which cancer cells disrupt apoptosis signaling [11]. This mechanism has been previously reported in HNSCC [12, 13], and upregulation of BCL-2 has been shown to be associated with cisplatin resistance and poor outcome in oropharyngeal squamous cell carcinoma [14, 15]. There have been limited studies examining BCL-xL and MCL-1 in HNSCC. Because anti-apoptotic proteins such as BCL-2, BCL-xL, and MCL-1 work in concert to inhibit apoptosis, the redundant role that these molecules play in HNSCC deserves further examination.

In the current study, we compare HNSCC tumors that were eradicated by chemoradiation to those that persisted or recurred locoregionally after treatment. Gene expression data were analyzed to identify transcripts that could differentiate these two groups, and BCL-xL was noted to be involved in several key networks among the genes identified. This led us to examine the efficacy of small-molecule targeting of BCL-xL in HNSCC. Treatment with ABT-263 (navitoclax), a small molecule inhibitor of BCL-2/BCL-xL, was effective in killing HNSCC cells at high doses, but had limited additive effect in combination with radiation or cisplatin. This led to examination of factors limiting the efficacy of BCL-xL/BCL-2 inhibition, specifically MCL-1 activity. MCL-1 expression was associated with resistance to radiation in HNSCC cells and found to increase in expression after treatment with ABT-263 (navitoclax). Co-inhibition of MCL-1 was required to optimize approaches targeting BCL-2/BCL-xL in HNSCC, resulting in synergistic activity.

## RESULTS

### Gene expression profile comparing treatment failures to responders

A cohort of patients with HNSCC were selected for the study based on the inclusion and exclusion criteria described in the methods section. The characteristics of the patient cohort are presented in Table 1. Patients with HNSCC who received radiation as a component of therapy and failed treatment (n = 23, with median time to failure = 15 months) were compared to patients who were treated with radiation or chemoradiation and remained free of locoregional recurrence at last follow-up (n = 35, median follow-up 41 months). For one subject in the failure group, gene expression data from both the primary tumor and the recurrent tumor were included in the analysis, as gene expression data was captured for both tumors. A comparison between characteristics for the failure group and the responder group, including primary tumor site, p16-assessment among oropharynx cancer patients, stage at diagnosis, and presence of lymph node disease, are presented in Table 1. Only disease primary site showed a statistically significant difference between groups, with a larger proportion of patients with oral cavity disease in the disease failure group (chi-square, p = 0.03).

**Table 1:** Patient and tumor characteristics

Variables	Failure Group		Responder Group		p-value
	N	%	N	%	
Total	23	100	35	100	
Site					
Larynx	7	30%	18	51%	0.03
Oropharynx	9	39%	15	43%	
p16+	4	17%	6	17%	
p16-	4	17%	7	20%	
unknown	1	4%	2	6%	
Oral Cavity	7	30%	2	6%	
Stage					
I, II	2	9%	2	6%	0.66
III, IV	21	91%	33	94%	
Nodal Status					
Positive	18	78%	29	83%	0.66
Negative	5	22%	6	17%	

**Figure 1:**
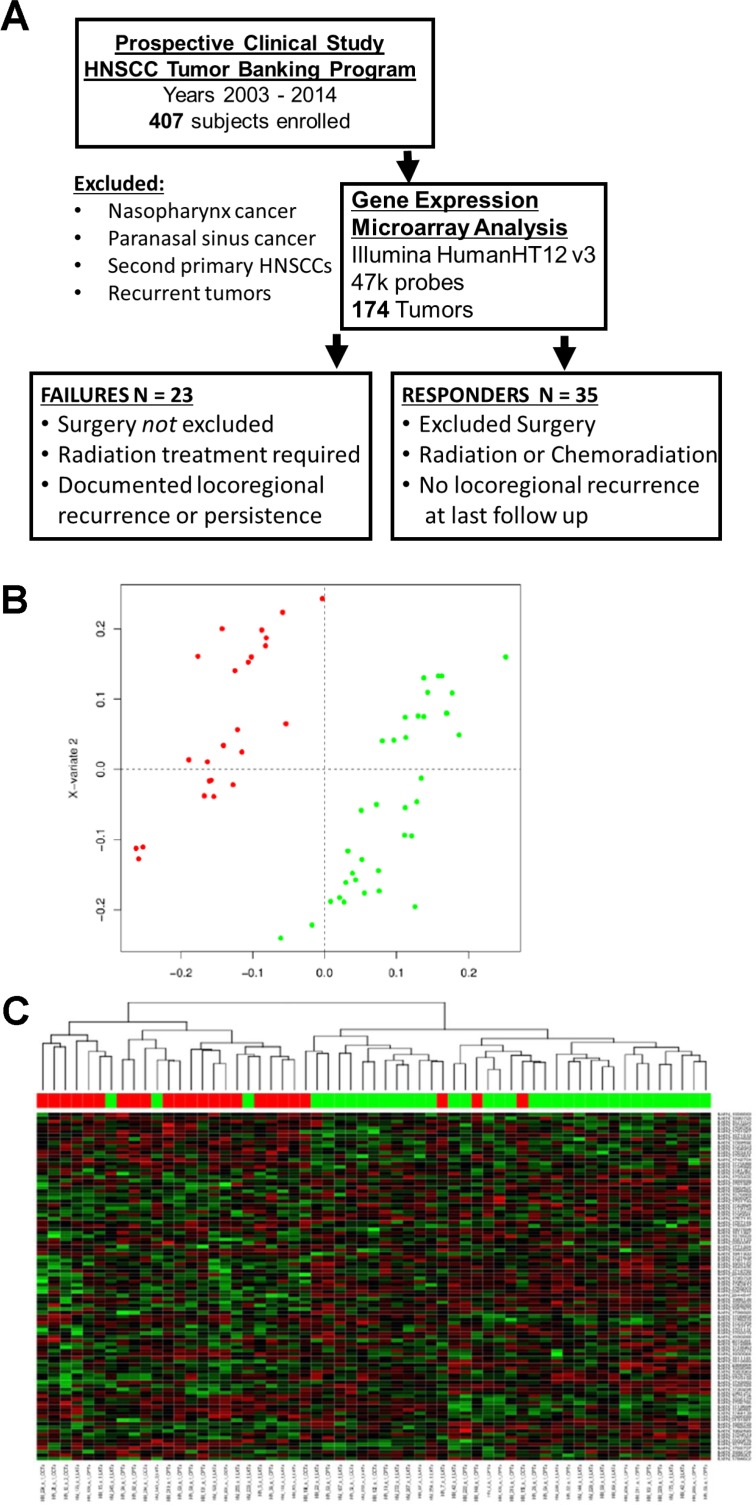
Patient cohort and gene expression data **(A)** Diagram describing the selection process for patients deemed *failures* and *responders* used for gene expression analysis. **(B)** Diagram of results from sparse Partial Least Squares-Discriminant Analysis (sPLS-DA). Red – *failure* cases, green – *responder* cases. **(C)** Heatmap of gene expression profiles from genes selected on the first component of the sPLS-DA model shows HNSCC treatment responders largely cluster separately from failures.

The gene expression profiles of tumors from patients with locoregional failure were compared to the profiles of tumors from responders using sPLS-DA (Figure 1B). The optimal sPLS-DA model had 100 features selected on each of two components, so nominally, 200 probesets (Supplementary Figure 1 - crossvalidation plot). Once duplicates and control probes were removed, 170 remained. Predicted genes/pseudogenes were removed, and 135 genes were ultimately identified that could be used to differentiate the two groups (Supplementary Table 1). A heatmap of the selected genes demonstrating segregation of the responder group and failure group is presented in Figure 1C. The identified genes were analyzed using IPA analysis. 73 canonical pathways were identified, and notably, BCL-xL was involved in 17 (23%) of these pathways. Based on this observation, BCL-family pro-survival molecules were selected for further study. The top pathways identified with IPA and networks involving BCL-xL are summarized in Supplementary Table 2. Since BCL-xL was identified as a key transcript involved in pathways that differentiated treatment-resistant from treatment-sensitive tumors, we proceeded to examine the role of BCL-xL and related family members in response to standard treatment and as potential treatment targets.

### Radiation and cisplatin response profiles of HNSCC cell lines

**Figure 2:**
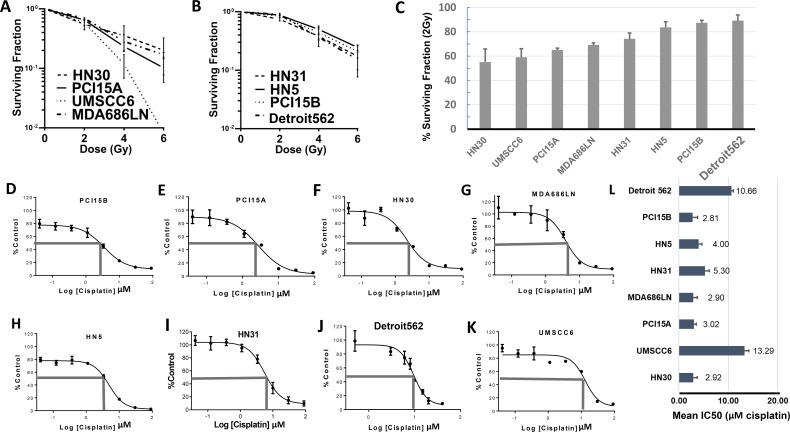
Baseline profiling of radiation and cisplatin response in HNSCC cell lines Results from clonogenic survival assays showing radiation sensitive **(A)** and radiation resistant **(B)** head and neck squamous cell carcinoma cell lines – average surviving fraction with standard error of the mean (S.E.M., error bars) is calculated from triplicate experiments; **(C)** Graphical representation of surviving fraction after 2 Grey dose of radiation (SF 2Gy), with cell lines arranged from most sensitive to most resistant; **(D–K)** Representative results from MTT assays of cisplatin in head and neck squamous cell cancer cell lines organized from most sensitive **(D–G)** to most resistant **(H–K)** to cisplatin. **(L)** Graphical representation of the average IC50, with S.E.M. (error bars) (MTT assay done in triplicate), cell lines arranged according to radiation sensitivity. There was no significant correlation between radiation sensitivity and IC50 dose for cisplatin.

Eight HNSCC cell lines (HN30, HN31, PCI15A, PCI15B, UMSCC6, MDA686LN, HN5, and Detroit562) were used to examine responses to radiation and cisplatin. Surviving fraction of cells after exposure to 2Gy, 4Gy, and 6Gy radiation were examined in clonogenic survival assays performed in triplicate. HNSCC cell lines demonstrated a range of survival to 2Gy radiation - from HN30: 55.1% (±10.7% Standard Error (SEM)) to Detroit562: 89.2% (±4.6% SEM). Data for more radiation sensitive cell lines (HN30, PCI15A, UMSCC6, and MDA686LN) are presented in Figure 2A, and more radiation resistant lines (HN31, HN5, PCI15B, and Detroit562) in Figure 2B. The cell lines were ranked by relative radiation sensitivity based on responses to 2Gy radiation (Figure 2C). Data for Figure 2A, 2B, and 2C are also included in tabular form in Table 2. For the remainder of this paper where cell lines are listed, they remain in the order of relative radiation sensitivity (based on surviving fraction at 2 Gy) for ease of reference.

**Table 2:** Surviving fraction at 2Gy, 4Gy, and 6y radiation based on clonogenic survival assays for head and neck squamous cell carcinoma cell lines

Cell line	2Gy (%SF)	S.E.M.	4Gy (%SF)	S.E.M.	6Gy (%SF)	S.E.M.
HN30	55.1	±15.16	35.5	±23.12	20.1	±17.47
UMSCC6	59.1	±15.12	11.9	±7.80	0.8	±0.57
PCI15A	65.2	±2.31	23.3	±5.84	9.9	±6.36
MDA686LN	65.3	±1.40	32.4	±11.63	16.9	±2.10
HN31	74.2	±6.75	38.0	±17.89	17.3	±13.72
HN5	83.6	±6.62	44.0	±13.91	21.0	±6.92
PCI15B	87.4	±2.19	50.0	±13.91	24.5	±6.92
DET562	89.2	±6.53	37.4	±5.51	22.4	±7.27

Abbreviations: Gy (Gray); SF (surviving fraction); S.E.M. (standard error of the mean).

Cell viability assays using MTT (3-(4,5-dimethyl-thiazol-2-yl)-2,5-diphenyltetrazolium bromide) were also used to assess response of the 8 cell lines to cisplatin treatment. The half maximal inhibitory concentration (IC50) for cisplatin ranged from PCI15A: 2.81μM (standard deviation (SD) ±0.99) μM to UMSCC6: 13.29μM (SD ±0.88μM). Representative cell viability curves are presented in Figure 2D–2K, and average IC50 values from assays done in triplicate are presented in Figure 2L. Cisplatin response showed no significant correlation with response to radiation among these 8 cell lines (Pearson's r = −0.016, p = 0.97).

### BCL-2 and BCL-xL expression and response to standard treatment and ABT-263

**Figure 3:**
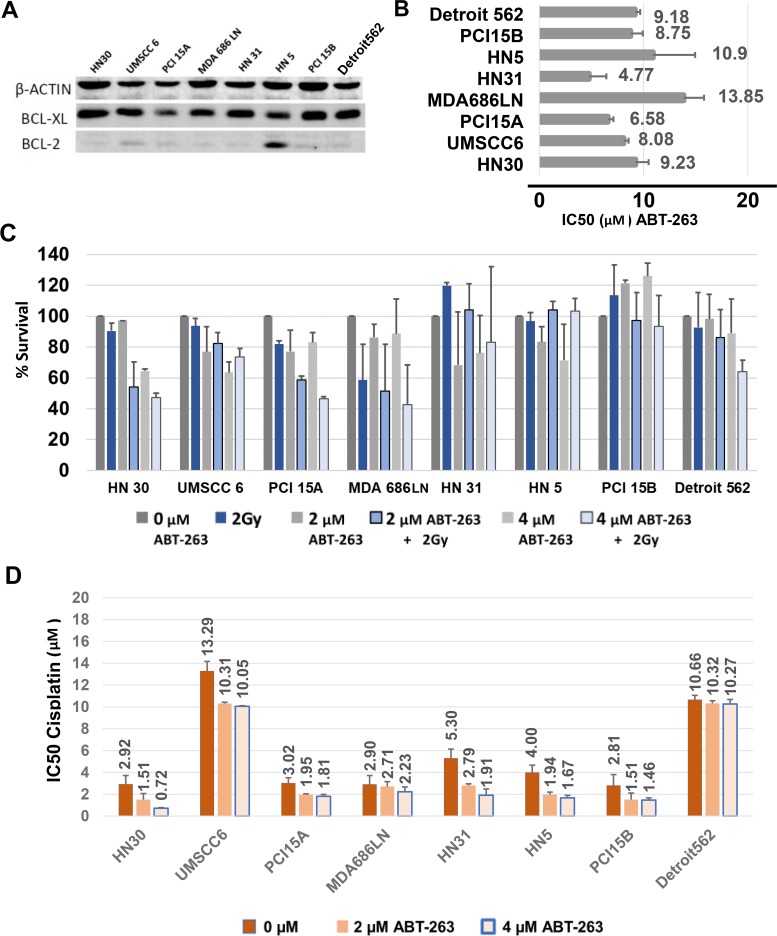
Baseline BCL-xL expression and inhibition of BCL-xL with ABT-263 (navitoclax) treatment of HNSCC cells, alone and in combination with radiation or cisplatin **(A)** Western blot demonstrating consistent high expression of BCL-xL and rare expression of BCL-2 in HNSCC cells. **(B)** Average IC50 values, with S.E.M. (error bars), after treatment with ABT-263 (navitoclax) assessed from cell viability assays using MTT. **(C)** Percent Survival calculated from clonogenic survival assays, with S.E.M. (error bars), after treatment of HNSCC cells with 2μM or 4μM ABT-263 (navitoclax) alone or in combination with 2 Gy radiation. **(D)** Average IC50 values, with S.E.M. (error bars), for cisplatin given in combination with 2μM and 4 μM ABT-263 (navitoclax).

*In vitro* studies were carried out to determine if BCL-xL, and related anti-apoptosis family members were associated with radiation and cisplatin response. The efficacy of targeting BCL-xL therapeutically was also examined. Baseline protein expression of BCL-2 and BCL-xL were assessed using Western Blot. BCL-xL was strongly and consistently expressed in all cell lines, while conversely, BCL-2 showed very weak or no expression in 7 cell lines: only HN5 demonstrated consistent high expression of BCL-2 (Figure 3A). There was no clear association between BCL-2 or BCL-xL basal expression and response to radiation or cisplatin.

ABT-263 (navitoclax) is a potent inhibitor of BCL-xL, with activity against BCL-2 and BCL-w [16]. The HNSCC cell line panel was treated with ABT-263 and responses were characterized via cell viability assays using MTT, tested in triplicate. Consistent responses to ABT-263 were noted in all cell lines tested at relatively high doses for each line (Figure 3B, Supplementary Figure 2). The IC50 to ABT-263 ranged from 4.77μM ±1.66μM (HN31) to 13.85μM ±1.95 μM (MDA686LN), with an average overall IC50 of 8.9μM. There was not a clear association noted between drug response in each line and baseline BCL-xL protein levels, radiation sensitivity, or cisplatin sensitivity.

The efficacy of ABT-263 in combination with 2Gy radiation was evaluated in clonogenic survival assays examining the panel of 8 HNSCC cell lines, performed in duplicate. A third trial was not performed because combination treatment was generally ineffective. 2μM and 4μM doses of ABT-263 were studied (Figure 3C). In general, ABT-263 (navitoclax) did not appear to radiosensitize HNSCC cells, and combining radiation with ABT-263 yielded only modest benefit in two lines (HN30, PCI15A).

The effect of combining cisplatin with ABT-263 was also studied using cell viability assays with MTT. We examined whether 2μM and 4μM doses of ABT-263 reduced the IC50 of cisplatin when given in combination. ABT-263 did decrease the IC50 of cisplatin in most cell lines (Figure 3D), however improvements were generally modest. For example, HN30 showed an approximately 4-fold decrease in the cisplatin requirement to achieve the IC50 after treatment with 4μM of ABT-263 (2.92μM ±0.80μM vs. 0.72μM ±0.05μM), while Detroit562 showed no change in the IC50 (10.66μM ±0.40μM vs. 10.27μM ±0.41μM).

Overall, the effect of inhibition of BCL-xL/BCL-2/BCL-w with ABT-263 in combination with standard therapy for HNSCC was not robust, which led to an exploration of possible mechanisms of resistance to ABT-263.

### MCL-1 expression in HNSCC cell lines and the HNSCC patient cohort

**Figure 4:**
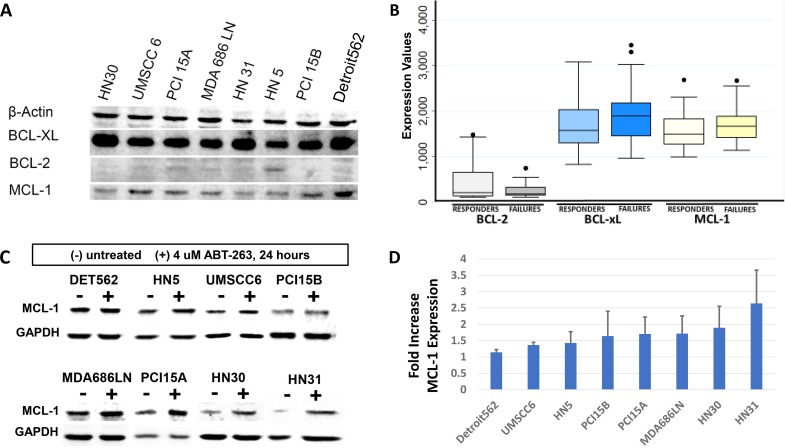
Evaluation of MCL-1 expression in HNSCC **(A)** Western blot demonstrating baseline MCL-1 expression in HNSCC cell lines, compared to BCL-xL and BCL-2 expression as demonstrated previously. Cell lines most resistant to radiation (HN5, PCI15B, Detroit562) demonstrate high MCL-1 expression. **(B)** Box and whisker plot representing gene expression transcript levels for BCL-2, BCL-xL, and MCL-1 as measured on gene expression microarrays in the HNSCC patient cohort, stratified by “responders” and “failures”. Lines represent median average transcript levels; Boxes represent 25^th^ and 75^th^ percentile, whiskers represent 2^nd^ and 98^th^ percentile. Dots represent outlying data points. **(C)** Western blot demonstrating MCL-1 protein expression at baseline, and 24 hours after treatment with 4μM ABT-263 (navitoclax). **(D)** Quantification of MCL-1 expression shows consistent increases of average MCL-1 expression, with standard deviation (error bars), after treatment with ABT-263 (navitoclax).

ABT-263 targets BCL-xL and BCL-2, but does not target the MCL-1 protein, another BCL-2 family pro-survival molecule. We therefore examined MCL-1 expression across the panel of 8 HNSCC cell lines using Western Blot (Figure 4A). Notably, the cell lines with highest MCL-1 expression were among those most resistant to radiation. In addition, we also examined expression of the BCL-2, BCL-xL, and MCL-1 transcript levels in the study patient cohort. The transcript expression levels in patient tumors mirrored protein expression observed in HNSCC cell lines – ie. expression of BCL-2 was very low, while expression levels of BCL-xL and MCL-1 were comparably high (Figure 4B). The expression values were compared between the treatment “failure” and treatment “responsive” groups. The expression levels of BCL-xL and MCL-1 were both elevated in tumors that failed treatment compared to those that responded, however these differences did not reach a p<0.05 threshold for significance. For BCL-xL, median expression among responders was 1579 (IQR 1304, 2030), compared to failures (1900, IQR 1454, 2175) (p = 0.08). For MCL-1, responders was 1489 (IQR 1271, 1832), compared to failures (1665, IQR 1418, 1888) (p = 0.23).

It was also hypothesized that treatment with ABT-263 (navitoclax) would result in upregulation of MCL-1 expression. MCL-1 expression was evaluated in the panel of 8 cell lines 24 hours after treatment with 4μM of ABT-263. MCL-1 was indeed noted to increase in all cell lines after ABT-263 (navitoclax) treatment (Figure 4C, Figure 4D). Paired *t*-test comparing MCL-1 levels between untreated and treated samples showed that MCL-1 was significantly increased after treatment with ABT-263 (p=0.04). BCL-2 and BCL-xL levels were also examined on western blot before and after treatment of 4 μM ABT-263. BCL-2 was not expressed in several lines, both before and after treatment, and no consistent increase in expression was observed. BCL-xL was significantly increased after treatment with ABT-263 (p=0.01).

### Effect of dual inhibition of BCL-xL and MCL-1

**Figure 5:**
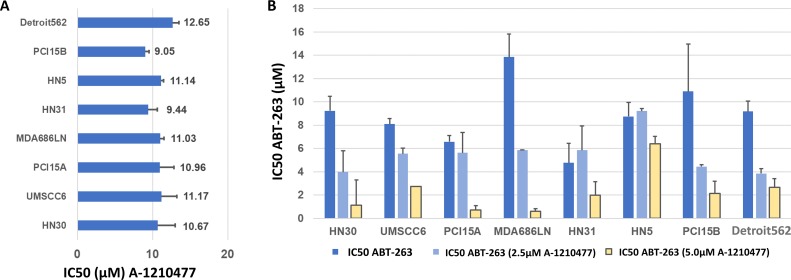
Inhibition of MCL-1 in HNSCC cells with A-1210477, alone and in combination with ABT-263 (navitoclax) **(A)** Average IC50 values, with S.E.M. (error bars), for A-1210477 in HNSCC cell lines. **(B)** Average IC50 values, with S.E.M. (error bars), for ABT-263 (navitoclax) in combination with 2.5 μM and 5μM A-1210477. Note that baseline IC50 values for A-1210477 **(A)** vary slightly from control values **(B)** as these were carried out as independent experiments with triplicate data for each.

In addition, we tested the response of the HNSCC cell lines to a recently-described [17] selective small molecule inhibitor against MCL-1: A-1210477 using MTT assays. Responses to this agent were consistent in all lines tested, with IC50 dosages ranging between 9.44±1.19μM (HN31) to 12.65μM ±0.84μM (Detroit 562) (Figure 5A, Supplementary Figure 3).

Next, the efficacy of A-1210477 was examined in combination with ABT-263 using MTT assays. HNSCC cell lines were treated with 2.5μM and 5μM doses of A-1210477 to determine if this resulted in a dose reduction required to achieve the IC50 with ABT-263. The assays were performed in triplicate. The IC50 of ABT-263 was substantially reduced in all cell lines tested (Figure 5B). The IC50 dose of ABT-263 was reduced on average 7-fold when combined with 5μM of A-1210477, ranging from 1.4-fold (HN5) to 22.3-fold (MDA686LN).

**Figure 6:**
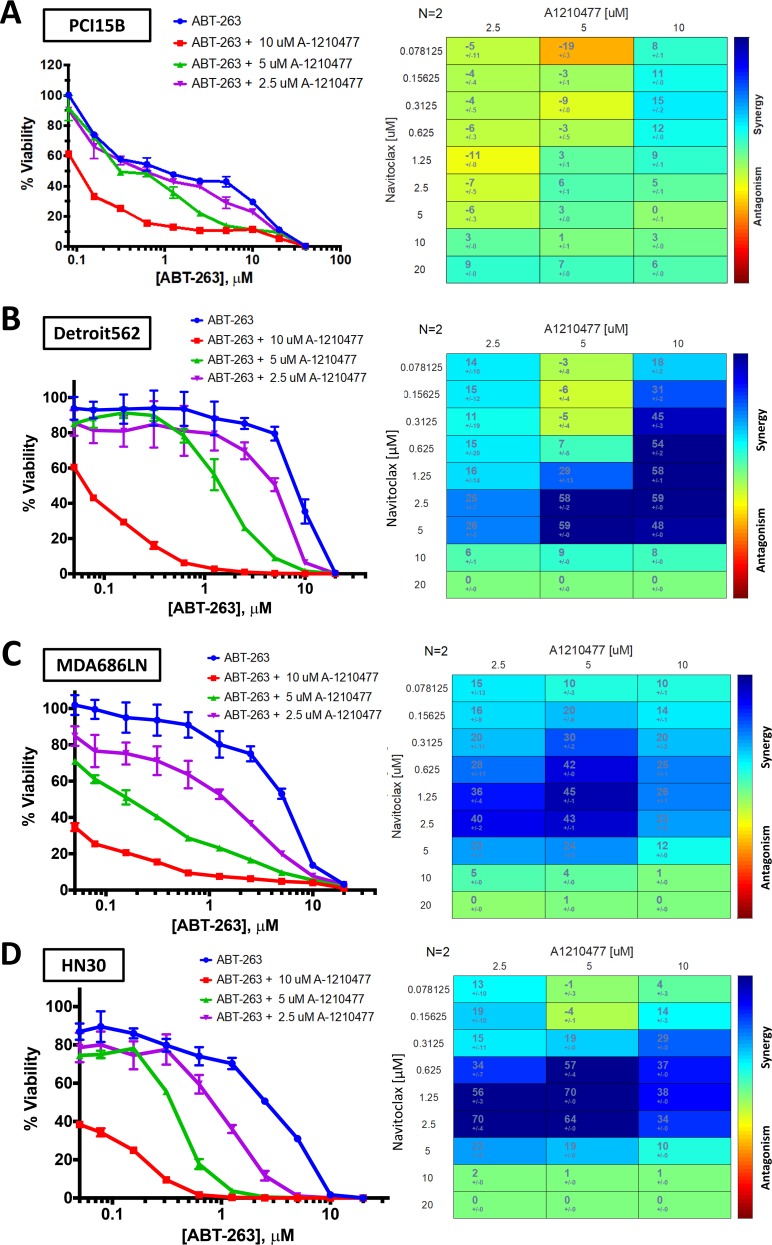
Bliss Independence analysis to evaluate synergistic activity between ABT-263 (navitoclax) and A-1210477 Cell viability curves and Bliss Independence analysis scores after treatment with ABT-263 (navitoclax) and A-1210477 across varying doses in PCI15B **(A)**, Detroit 562 **(B)**, MDA686LN **(C)**, and HN30 **(D)**.

It was suspected that the drug combination of ABT-263 and A-1210477 was synergistic based on results of the 8 cell line panel. Four of the cell lines (PCI15B, Detroit 562, MDA686LN, HN30) were examined using cell viability assays, with expanded dose ranges to allow for Bliss Independence synergy analysis. These were performed in duplicate as they were confirmatory to the MTT assays. Bliss Independence analysis showed there was synergy observed for the drug combination in each line (Figure 6). Results in PCI15B were modest with synergy noted when 10μM of A-1210477 was administered, however the other three lines demonstrated synergy when 2.5μM of A-1210477 was administered with ABT-263 (navitoclax).

## DISCUSSION

It remains unclear why some HNSCC tumors respond exquisitely to cisplatin and radiation, while others fail. We used an analysis of gene expression data to differentiate between HNSCCs that recur or persist after treatment versus those that do not. Other studies have taken a similar approach. Ginos and colleagues [18] compared gene expression profiles from microarray data between 41 HNSCC samples and 13 normal controls to identify a signature that was specific to HNSCC tumor specimens, and they also used the expression data to identify genes that could differentiate HNSCCs that recurred versus those from patients who had not exhibited a recurrence. Of note, this study included several specimens that had been harvested after primary treatment and recurrence, while only 7 specimens in the recurrence group were from pre-treatment biopsies. Another study by Chung, et al. [19], examined 60 HNSCC samples using gene expression microarrays, and gene expression profiles for these tumors were segregated into four groups using unsupervised methods. When examining the association of these profiles with clinical parameters, it appeared that one subgroup demonstrated improved recurrence-free survival. A follow up study by the same investigators [20] identified a 75-gene signature that could differentiate HNSCC tumors that recurred from those that did not among a test set of 40 samples from 29 patients. The predictive ability of this signature was then validated on tumors from an additional cohort of 60 patients.

While these described studies aimed to generate gene expression profiles that could stratify tumors into groups with high or low risk of recurrence, none of these profiling methods have become clinically relevant, to date. Our study attempted to use the information we gained from gene expression analysis to identify a potential strategy to better treat HNSCC tumors with high risk of treatment failure. Instead of focusing on a single gene or single pathway, we hypothesized that a strategy that would disrupt *several* key signaling networks identified in our analysis would prove effective. To our knowledge, we are the first to consider this approach based on a gene expression discovery analysis.

Our investigation led us to focus on BCL-xL and other BCL-2 family proteins in HNSCC. Several other groups have examined the relationship between BCL-xL and BCL-2 in HNSCC and responses to treatment. Work by Bauer and colleagues demonstrated that patients with laryngeal SCC who express low levels of BCL-xL demonstrate better responses to chemotherapy and chemoradiation compared to patients with high BCL-xL expression, which was recapitulated in a HNSCC cell line model examining cisplatin-resistant cells [12]. In a similar report, correlative studies from an organ-preservation trial demonstrated that high BCL-xL expression as a component of a biomarker panel was associated with poor outcomes [21]. These data generally support our findings that BCL-xL expression is an important factor differentiating HNSCC tumors that either respond or fail locoregionally after chemoradiation. BCL-2 has also been examined in HNSCC. Recent work has demonstrated that BCL-2 expression conveyed cisplatin resistance in HNSCC cell lines [14], and was independently associated with poor outcome among patients with oropharyngeal SCC after adjusting for known prognosticators, such as HPV-status, in a multivariable model [15]. Both our patient data and *in vitro* work suggest that BCL-xL expression is expressed at higher levels than BCL-2, and that BCL-xL (and not BCL-2) is associated with treatment response. Our *in vitro* data also suggest that MCL-1 expression may be an important factor associated with resistance to radiation. Large prospective studies are required to validate whether immunohistochemical expression of BCL-xL, BCL-2, and MCL-1 in pre-treatment biopsy specimens on patients with HNSCC is associated with response to radiation/chemoradiation.

ABT-737, A BH3-mimetic small molecule that targets the BCL-2 family pro-survival proteins BCL-2, BCL-xL, and BCL-w, was first described in 2005. This drug showed anti-tumor activity in both *in vitro* and *in vivo* models, and also enhanced the effects of both cytotoxic chemotherapeutics and radiation [22]. ABT-263 (Navitoclax), an orally bioavailable version of ABT-737, was first described in 2008, and showed preclinical activity in both B-Cell lymphoma and small cell lung cancer cells [16]. ABT-263 (navitoclax) has been studied in phase I and II trials, and the efficacy of ABT-263 (navitoclax) in solid tumors has been largely disappointing, both as a single agent [23, 24] and in combination with other chemotherapeutics, including gemcitabine, carboplatin/paclitaxel, irinotecan, and erlotinib [25–28]. These studies collectively suggest that redundant anti-apoptotic mechanisms limit the efficacy of targeting BCL-2 and BCL-xL, which is consistent with our *in vitro* findings. The experience treating patients with HNSCC across all of these early studies has been very limited.

Therapeutics targeting BCL-2 pro-survival proteins have been tested preclinically in HNSCC. (-)-Gossypol, a BH3-mimetic which inhibits BCL-xL and BCL-2, has been studied in HNSCC cell lines, and (-)-gossypol demonstrated improved efficacy compared to cisplatin and induced apoptosis in cisplatin resistant HNSCC lines [29]. A study published in 2007 demonstrated that BH3-mimetic peptides targeting BCL-xL and BCL-2 could induce apoptosis in HNSCC cell lines [30]. The same group studied ABT-737 in HNSCC cells. Similar to the findings in our study, single-agent targeting of BCL-xL/BCL-2 had limited efficacy, while ABT-737 when applied in combination with cisplatin and etoposide appeared to have a synergistic effect and enhanced apoptosis measured by Annexin V staining and caspase cleavage [31]. Interestingly, this study showed that the cytotoxic agents resulted in down-regulation of MCL-1. Another study demonstrated that obatoclax, a proposed pan-BCL-2 inhibitor, was effective in inducing apoptosis and killing HNSCC cells [32]. However, obatoclax's mechanism of action has recently been challenged, suggesting it activates apoptosis through indirect mechanisms and does not specifically inhibit BCL-2 proteins [33]. A recent study has also demonstrated that resistance to a mammalian target of rapamycin (mTOR) inhibitor could be overcome with BCL-2 inhibition via ABT-737 treatment in a HNSCC cell line model [34]. While previous studies have examined the *in vitro* efficacy of BCL-2/BCL-xL inhibitors in HNSCC, both as single agents and in combination with chemotherapy, our study presents the examination of ABT-263 (navitoclax) alone and in combination with cisplatin in the largest number of HNSCC cell lines reported to date. Our study is also the first to examine the combination of ABT-263 (navitoclax) with radiation therapy in HNSCC, and only the second report we could find in any tumor type. Our comprehensive evaluation led us to draw generalizable conclusions for HNSCC, specifically that targeting BCL-xL and BCL-2 alone was effective, but did not result in consistent synergy when combined with radiation and/or cisplatin.

The limited activity of BH3-mimetics in solid tumors, as well as our data showing that high doses of ABT-263 were necessary to achieve a response in HNSCC cells, suggest that there are redundant mechanisms that inhibit apoptosis in cancer cells. Because BCL-2 and BCL-xL inhibitors fail to target MCL-1, MCL-1 expression is an obvious potential mechanism by which cancer cells resist treatment with ABT-263, and this mechanism has been demonstrated in some cancer models [35, 36]. MCL-1 expression has been described in HNSCC [37], and our review of the literature only identified one brief report suggesting MCL-1 expression was associated with response to treatment in HNSCC [38]. Our examination of MCL-1 gene expression indeed showed that MCL-1 transcript levels are generally high in HNSCC tumors. Additionally, high MCL-1 expression *in vitro* seemed to correlate with radiation resistance. It should be noted however, that it was BCL-xL that was identified in our gene expression evaluation, and not MCL-1, that appeared to be associated with locoregional control. It is not surprising that certain gene expression transcripts are useful biomarkers because of their consistent association with certain disease phenotypes (e.g. radiation resistance) and prognosis, while other molecules are important for treatment selection, but not necessarily predictive biomarkers.

MCL-1 specific inhibitors have been discovered very recently. The first report on A-1210477 was published in 2015 by Leverson and colleagues [17] in a study that demonstrated *in vitro* activity as a single agent, and synergistic effect when combined with ABT-263 when applied to several cancer cell lines. Phillips and colleagues demonstrated that resistance to BCL-2 inhibition with venetoclax in non-Hodgkin's lymphoma cells could be overcome with co-treatment with A-1210477, resulting in synergy between these two drugs [39]. The interplay between MCL-1 activity and response to BH3-mimetics has had limited evaluation in HNSCC. As mentioned above, Li et al., demonstrated that treatment with cytotoxic agents cisplatin and etoposide led to reduced MCL-1 expression in 3 HNSCC cell lines, which perhaps influenced synergistic effects when combined with ABT-737. In our study, we did not observe synergy between cisplatin and ABT-263, however the required dose of cisplatin to achieve the IC50 was reduced in most cell lines. In our panel of HNSCC, it appeared that MCL-1 was most closely correlated with radiation resistance compared to BCL-2 or BCL-xL expression, and combining inhibition of MCL-1 with ABT-263 demonstrated consistently improved efficacy, and synergy based on Bliss Independence analysis.

Our study represents a preliminary look at the complex interplay between apoptosis signaling molecules in HNSCC, and the authors recognize several limitations of our report. First, we acknowledge that the cohort of patients examined in our study is heterogenous, and that the definitions we applied for failure and response to treatment is perhaps unconventional. However, we placed careful restrictions and definitions to the discovery set in order to focus on the biology of resistance to non-surgical treatment. We also recognize that evidence of treatment responses in an *in vivo* model would best support our findings, and these studies are planned. To our knowledge, this study is the first to examine the combination of ABT-263 with an MCL-1 inhibitor in HNSCC. A-1210477 was selected as it was the earliest available MCL-1 inhibitor available for the studies in this report. This agent cannot be used *in vivo* due to the pharmacokinetic profile of this molecule [40], but other options now exist for inhibition of MCL-1. A recent study reported that afatanib, a dual EGFR and HER2 inhibitor, decreases MCL-1 expression in HNSCC cells [41]. Very recently a new MCL-1 inhibitor, S63845, was described and demonstrated both *in vitro* and *in vivo* efficacy against MCL-1 dependent myeloma, leukemia, and lymphoma cells [42]. This report also examined effects of this inhibitor with several other chemotherapeutic agents in solid tumor cell lines. Based on the data we have presented here, the combination of improved and clinically applicable MCL-1 inhibitors, such as S63845, with drugs targeting BCL-xL/BCL2, is a logical and potentially effective next step for *in vivo* studies of HNSCC.

In conclusion, expression of the BCL-2 family prosurvival molecule BCL-xL was a component of several gene networks identified after profiling treatment-resistant and treatment-sensitive HNSCC tumors. Inhibition of BCL-xL with ABT-263 (navitoclax) demonstrated consistent efficacy in HNSCC cells, but at relatively high doses. MCL-1 expression correlated with radiation resistance in HNSCC cells, and inhibition of MCL-1 with A-1210477 enhanced response to ABT-263 (navitoclax). Increasing clinical experience with BH3-mimetic agents, and newly characterized agents targeting MCL-1 may lead to novel and effective strategies targeting HNSCC.

## MATERIALS AND METHODS

### Patient cohort

Patients with newly diagnosed HNSCC were enrolled on an ongoing IRB-approved prospective cohort study and tumor banking program at our institution, as previously described [43, 44]. For this study, patients were enrolled and followed between 2002 – 2014. After obtaining written consent for participation, histologically confirmed HNSCC tumors were obtained by biopsy or surgical resection from patients undergoing treatment at Montefiore Medical Center in Bronx, New York. The specimen submitted for RNA extraction was procured from tissue deemed to be grossly viable tumor (areas of normal mucosa or gross necrosis were avoided) by the operating surgeon and/or pathologist. Tumors were snap frozen in liquid nitrogen within 30 minutes of procurement. Each tumor was assessed by a clinical pathologist to confirm diagnosis of HNSCC, and to measure percent tumor of each sample on a representative histologic section using hematoxylin and eosin staining. Details regarding tumor acquisition, pathologic confirmation and tumor content have been previously published [45, 46]. Patients were selected for this study by identifying patients who failed after treatment compared to those who remained free of locoregional recurrence after treatment according to the following criteria:

#### Inclusion criteria

Patients were required to have been diagnosed with squamous cell carcinoma with a primary tumor site in the upper aerodigestive tract. All patients were required to have undergone diagnostic biopsy and/or initial treatment for HNSCC at Montefiore Medical Center. Cases were defined as *treatment failures* if they received radiation or chemoradiation to treat their disease, and subsequently failed treatment with persistent or recurrent local (primary site) or regional (neck lymph node) disease in the treated field. Patients who also received surgery with subsequent radiation or chemoradiation were *included* among the *treatment failure* group, as locoregional failure in these cases were considered disease that resisted non-surgical therapy. Patients were defined as *treatment responders* if they received *primary* radiation or chemoradiation and experienced a complete response, with no documented local or regional recurrence at the last follow up at the time of analysis.

#### Exclusion criteria

Of note, subjects who received surgical resection as a component of their primary treatment were *not included* in the *treatment responder* group, in order to enrich this group for those tumors with complete response to and no recurrence after non-surgical treatment.

Patients with non-squamous cell cancer histology (eg. salivary gland carcinoma), and patients with primary tumors located in sites other than the upper aerodigestive tract (eg. cutaneous cancers) were excluded. Patients with primary cancer of the nasopharynx and paranasal sinuses were also excluded. Also, patients who underwent treatment for a previous head and neck cancer (ie. enrolled at diagnosis of a second or subsequent primary HNSCC), and those who were enrolled at the time of HNSCC recurrence were also excluded.

Figure 1A illustrates the selection process for inclusion and exclusion in the discovery analysis.

### Gene expression analysis

Total RNA was extracted from tumor tissue using TRIzol™ by a standardized protocol (Invitrogen™, Carlsbad, CA). RNA was collected by alcohol precipitation and quantitated for microarray analysis. Quality control was performed on selected samples, checking for integrity of RNAs using an Agilent 2100 bioanalyzer (Agilent, Santa Clara, CA) and RNA pico chips as described by the manufacturer. Total RNA (500 ng) was amplified and biotin labeled with the Illumina^®^ TotalPrep™ RNA Amplification Kit (Ambion^®^, Austin, Texas). Global expression was analyzed by RNA hybridization to the Illumina^®^ HumanHT-12-v3 Expression BeadChip (Illumina^®^, San Diego, California). Probes were matched to known genes and alternative splice variants using the RefSeq database release 17 (https://www.ncbi.nlm.nih.gov/refseq/) and UniGene build 188 (https://www.ncbi.nlm.nih.gov/unigene). Controls for each RNA sample were used to confirm RNA quality, biotin labeling success, hybridization stringency, and signal levels.

### Bioinformatic analysis

Microarray expression values were quantile normalized within BeadStudio (Illumina^®^) prior to analysis. Expression data were batch corrected using the ComBat function from the sva R package [47]. Supervised analysis using sparse Partial Least Squares-Discriminant Analysis (sPLS-DA) [48] was carried out to identify a set of gene expression biomarkers that differentiated the failure and responder groups. The sPLS-DA model was tuned using different combinations of parameters, namely, number of model components (1-10) and number of selected features (probesets) on each component (100-500). Model validation was carried out using leave-one-out crossvalidation (LOOCV) with the lowest predictive error being achieved using a 2-component model and selecting 100 features on each component. Genes identified as differentiating these two groups were analyzed using Ingenuity^®^ Pathway Analysis (IPA) (QIAGEN, Hilden, Germany).

### HNSCC cell culture

HNSCC cell lines HN30, HN31, PCI15A, PCI15B, UMSCC6, MDA686LN, and HN5, were obtained from a repository maintained by Dr. Jeffrey N. Myers, MD, PhD at the University of Texas, MD Anderson Cancer Center, with some cell lines requiring the following permissions (HN30, HN31 – John Ensley, MD, Wayne State University; PCI-15A, PCI15B – Jennifer Grandis, MD, University of Pittsburgh; UMSCC6 – Thomas Carey, University of Michigan; MDA686LN – Peter Sacks, MD, New York University/University of Texas MD Anderson Cancer Center). The Detroit562 cell line was acquired from the American Type Culture Collection. All cells were maintained in Dulbecco's Modified Eagle's Medium (DMEM) supplemented with 10% Fetal Bovine Serum (FBS), nonessential amino acids, sodium pryuvate and 1% antibiotic — penicillin/strepomycin. Cells were incubated at 37°C, 5% CO_2_. These 8 HNSCC cell lines were used in all of the *in vitro* assays described. Stock samples for all cell lines used were authenticated with short tandem repeat (STR) genotyping. All experiments were performed within 20 or less passages for each line.

### Clonogenic survival assays (γ-irradiation)

Cells were seeded onto 6-well plates at densities of 800–1600 cells/well, 24 hours before treatment. At 24 hrs cells were treated with γ-irradiation (Cesium-137, 2Gy/min) at indicated doses and returned to 37°C incubation. After 5 days media was replaced with fresh media, and cells were allowed to continue growth. Colony growth continued to be monitored up to 5-14 days post γ-irradiation. At the time of harvest, media was removed and cell clones were washed, fixed, and stained with 0.25% Cresyl Violet. The results were quantified with ImageJ software [49]. Plating efficiency was optimized for each cell line, and surviving fractions were calculated per standard methods [50]. To establish radiation or combination drug-radiation sensitivity, assays were carried out in triplicate.

#### Clonogenic survival assays combining ABT-263 treatment and radiation

Cells were seeded into 6-well plates as described above. Cells were pre-treated with ABT-263 for 4 hours before γ-irradiation (Cesium-137, 2Gy/min, JL Shepherd Mark I Model 68 Irradiator, JL Shepherd and Associates, San Fernando, CA). Harvest and survival calculations were carried out as described above. Experiments were performed in duplicate and not repeated a third time after preliminary results demonstrated little benefit with combination treatment.

### Western blot

Cells grown *in vitro* were washed with PBS, scraped from cell culture plates, and lysed using standard RIPA buffer. Protein concentrations of cell lysates were determined by Lowry Protein Assay (Bio-Rad Laboratories, Hercules, CA). Equal amounts of proteins were loaded onto SDS-PAGE gels, separated by electrophoresis, and transferred onto PVDF membranes. Membranes were washed in TBS-T and blocked in 1% BSA at room temperature for 1 hour or in 4°C overnight. The membrane was probed overnight at 4°C with primary antibody. Then it was washed and incubated with appropriate secondary antibody. Membranes were probed using the following antibodies: BCL-2, BCL-xL, MCL-1 (Cell Signaling Technology, Danvers, MA), each at 1:500 dilution and Beta-Actin as a loading control protein (Santa Cruz Biotechnology, Dallas, Tx) at 1:10,000 dilution. Following fluorescent secondary antibody incubation at 1:2500 dilution, immunoreactive proteins were visualized with the LI-COR^®^ Odyssey^®^ FC Imaging System. Protein band quantification was determined with image studio software (LI-COR^®^ Biosciences, Lincoln, NE). All protein quantification experiments were carried out on a minimum of three representative western blots.

### Cell viability assays (MTT)

All cell lines were plated on 96-well plates at 1,500 cells/well (UMSCC6 at 4,000 cells/well). Cells were incubated for 24 hours before drug addition. Incubation time was maintained between 48 - 72 hours for each condition. Conditions were optimized for each condition. Cell death was generally more rapid when cisplatin treatment was included. Specifically, incubation time for Cisplatin alone was 48hrs, ABT-263 (navitoclax) alone was 72 hrs, Cisplatin and ABT-263 (navitoclax) was 48 hrs, A-1210477 was 72 hrs and ABT-263 (navitoclax) plus A-1210477 was 72 hrs All drugs were initially dissolved in dimethyl sulfoxide (DMSO), and thus control (untreated) wells were maintained in corresponding dilutions of dissolvent DMSO. After drug incubation, MTT 3-(4,5-dimethylthiazol-2-yl)-2,5-diphenyltetrazolium bromide) was added until visualization of formazan crystals, approximately 180 minutes. All media was then aspirated from wells, and the cells were lysed in 100 μL of DMSO on an orbital pulse shaker. Approximate viability was determined with Benchmark Plus™ Microplate Spectrophotometer System (Bio-Rad, Laboratories, Hercules, CA) at 570 nm. The percentage of growth rates were calculated relative to the untreated control and were graphed as a function of the log_10_ concentration. IC50 values were calculated by non-linear regression four-parameter logistics curve analysis with GraphPad Prism (GraphPad Software, La Jolla, CA). All MTT assays were repeated in triplicate.

### Cell viability assays for Bliss Independence analysis

HNCC cells (1500 cells/well) were seeded in 96-well white plates. Cells were incubated with serial dilutions of ABT-263 (20 mM to 0.078 mM) and co-treated with A1210477 at the indicated concentrations. Control (untreated) wells were maintained in corresponding dilutions of dissolvent DMSO. Cell viability was assayed after 72 hours treatment using the Cell Titer-Glo (Promega, Madison, WI), according to the manufacturer's protocol. Luminescence was measured using the TECAN M200 microplate reader. Viability assays were performed in duplicates and the data normalized to vehicle-treated control wells. IC50 values were determined by nonlinear regression analysis using Prism software (GraphPad Software, La Jolla, CA). The Bliss Independence combination synergy was assessed using the Combenefit platform as previously described in Veroli et al. Bioinformatics 2016. [51]

### Statistical analysis

Bioinformatic analysis carried out on the patient cohort is described in a preceding section. Descriptions and references for calculations for plating efficiency/surviving fraction, IC50 calculations, and Bliss Independence analysis are described in their respective sections above. Mean averages and standard deviation (SD) or standard error of the mean (SEM) were used to present normally distributed data, where appropriate (i.e. average surviving fraction after radiation, average IC50 for MTT assays, average fold increase in MCL-1 expression on Western blot). Median average and interquartile ranges (IQR, 25%, 75%) were calculated for gene expression levels of BCL-xL, MCL-1 and BCL2, and comparisons between responders and failure subjects were carried out using Mann-Whitney *U* tests. Paired *T*-tests were used to compare expression of BCL-xL and MCL-1 in HNSCC cell lines before and after treatment with ABT-263 (navitoclax). A two-sided p-value < 0.05 was considered statistically significant for all statistical calculations.

## SUPPLEMENTARY MATERIALS FIGURES AND TABLES







## Abbreviations

AJCC: American Joint Committee on Cancer
BSA: bovine serum albumin
DMEM: Dubelcco's modified Eagle's medium
DNA: deoxyribonucleic acid
HNSCC: head and neck squamous cell carcinoma
Gy: Gray
HPV: human papillomavirus
hrs: hours
IC50: half maximal inhibitory concentration
IQR: interquartile range
IPA: Ingenuity Pathway Analysis
LOOCV: leave one out cross-validation
MTT: 3-(4,5-dimethylthiazol-2-yl)-2,5-dipheny- ltetrazolium bromide
ng: nanogram
nm: nanometer
PBS: phosphate buffered saline
PVDF: polyvinylidene fluoride
RIPA: radioimmunoprecipitation assay
RNA: ribonucleic acid
SCC: squamous cell carcinoma
SD: standard deviation
SDS-PAGE: sodium dodecyl sulphate-polyacrylamide gel electrophoresis
SEM: standard error of the mean
sPLS-DA: sparse partial least squares – discriminant analysis
μM: micromolar
TBS-T: tris-buffered saline - Polysorbate 20 (Tween 20)
μL: microliter
