# Supplementary material for: Optimal targeting of BCL-family proteins in head and neck squamous cell carcinoma requires inhibition of both BCL-xL and MCL-1
Source: Oncotarget. 2019 Jan 11;10(4):494–510. doi: 10.18632/oncotarget.26563 (PMC6355180; doi:10.18632/oncotarget.26563)
Supplement: Supplementary file 1 [file oncotarget-10-494-s001.pdf]

# Optimal targeting of BCL-family proteins in head and neck squamous cell carcinoma requires inhibition of both BCL-xL and MCL-1

## SUPPLEMENTARY MATERIALS

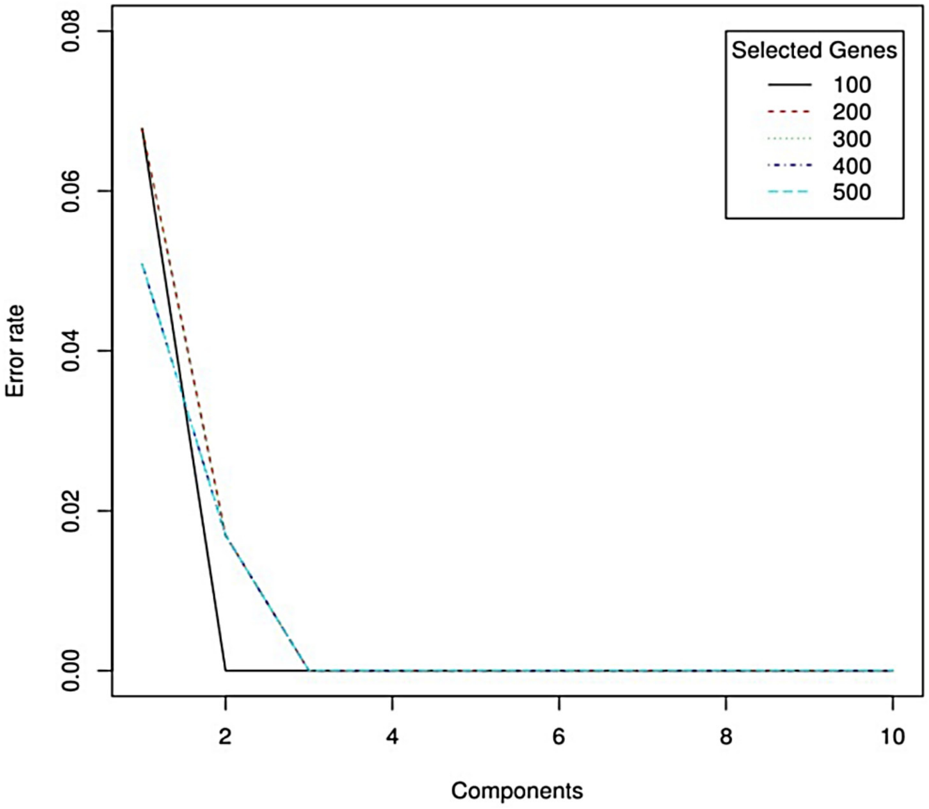


**Supplementary Figure 1: Crossvalidation plot demonstrating the predictive error of 2 component models using different numbers of selected features.** 100 specific features optimizes the 2 component model.

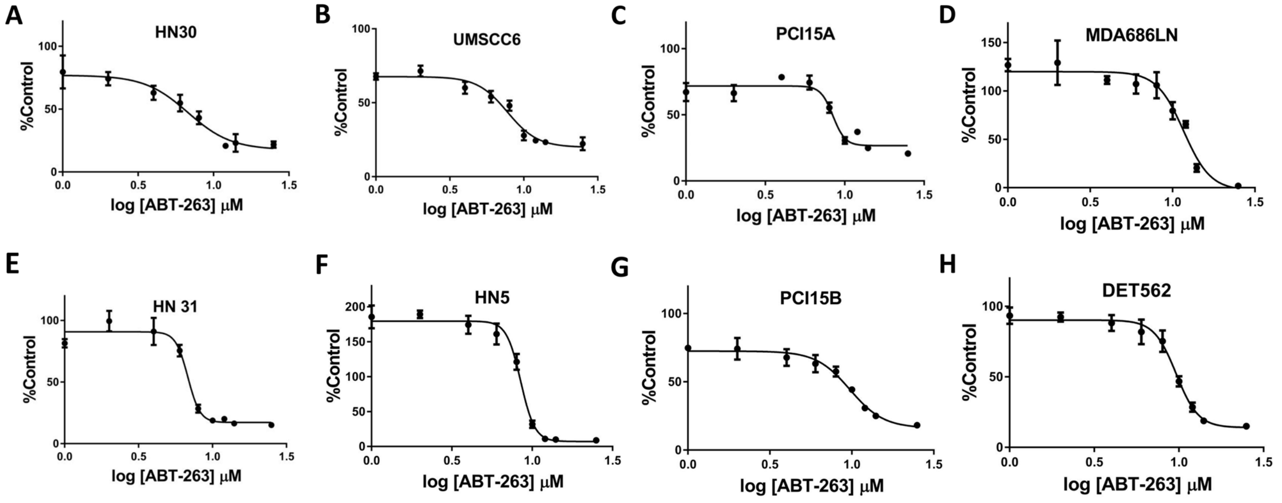


**Supplementary Figure 2: Representative results from MTT assays evaluating response of head and neck squamous cell cancer cell lines to ABT-263 (navitoclax).** ABT (navitoclax) for **(A)** HN30, **(B)** UMSCC6, **(C)** PCI15A, **(D)** MDA686LN, **(E)** HN31, **(F)** HN5, **(G)** PCI15B, and **(H)** Detroit562.

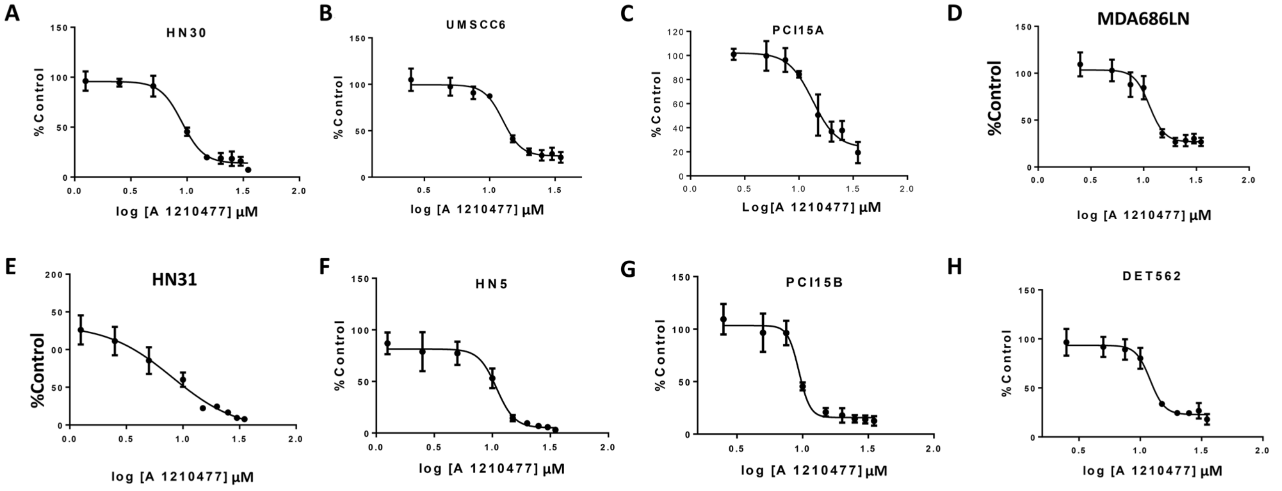


**Supplementary Figure 3: Representative results from MTT assays evaluating response of head and neck squamous cell cancer cell lines to A-1210477.** A-1210477 for **(A)** HN30, **(B)** UMSCC6, **(C)** PCI15A, **(D)** MDA686LN, **(E)** HN31, **(F)** HN5, **(G)** PCI15B, and **(H)** Detroit562.

**Supplementary Table 1: Annotated list of genes differentiating treatment-resistant from treatment-sensitive head and neck squamous carcinoma**

**See Supplementary File 1**

**Supplementary Table 2: Canonical pathways identified using Ingenuity Pathway analysis to evaluate 135 genes differentiating treatment-resistant from treatment-sensitive head and neck squamous cell carcinoma**

**See Supplementary File 2**
