# Supplementary material for: Optimal targeting of BCL-family proteins in head and neck squamous cell carcinoma requires inhibition of both BCL-xL and MCL-1
Source: Oncotarget. 2019 Jan 11;10(4):494–510. doi: 10.18632/oncotarget.26563 (PMC6355180; doi:10.18632/oncotarget.26563)
Supplement: Supplementary file 2 [file oncotarget-10-494-s002.docx]

**Supplementary Table 1: Annotated list of genes differentiating treatment-resistant from treatment-sensitive head and neck squamous carcinoma**

| **Symbol** | **Entrez Gene Name** | **Location** | **Type(s)** |
| --- | --- | --- | --- |
| ABL2 | ABL proto-oncogene 2, non-receptor tyrosine kinase | Cytoplasm | kinase |
| ACACB | acetyl-CoA carboxylase beta | Cytoplasm | enzyme |
| AGAP6 (includes others) | ArfGAP with GTPase domain, ankyrin repeat and PH domain 6 | Other | other |
| ANGEL2 | angel homolog 2 (Drosophila) | Nucleus | other |
| AP2S1 | adaptor-related protein complex 2, sigma 1 subunit | Cytoplasm | transporter |
| APBB2 | amyloid beta (A4) precursor protein-binding, family B, member 2 | Cytoplasm | other |
| ARF1 | ADP-ribosylation factor 1 | Cytoplasm | enzyme |
| ARMCX3 | armadillo repeat containing, X-linked 3 | Cytoplasm | other |
| BCL2L1 | BCL2-like 1 | Cytoplasm | other |
| BOLA2/BOLA2B | bolA family member 2 | Cytoplasm | other |
| C20orf166-AS1 | C20orf166 antisense RNA 1 | Other | other |
| C22orf31 | chromosome 22 open reading frame 31 | Other | other |
| C2orf49 | chromosome 2 open reading frame 49 | Other | other |
| CACNG2 | calcium channel, voltage-dependent, gamma subunit 2 | Plasma Membrane | ion channel |
| CANX | calnexin | Cytoplasm | other |
| CASP3 | caspase 3, apoptosis-related cysteine peptidase | Cytoplasm | peptidase |
| CD68 | CD68 molecule | Plasma Membrane | other |
| CDKL2 | cyclin-dependent kinase-like 2 (CDC2-related kinase) | Nucleus | kinase |
| CDKN1A | cyclin-dependent kinase inhibitor 1A (p21, Cip1) | Nucleus | kinase |
| CDKN2B | cyclin-dependent kinase inhibitor 2B (p15, inhibits CDK4) | Nucleus | transcription regulator |
| CHCHD7 | coiled-coil-helix-coiled-coil-helix domain containing 7 | Cytoplasm | other |
| CHRFAM7A | CHRNA7 (cholinergic receptor, nicotinic, alpha 7, exons 5-10) and FAM7A (family with sequence similarity 7A, exons A-E) fusion | Other | other |
| CLCN5 | chloride channel, voltage-sensitive 5 | Plasma Membrane | ion channel |
| CPEB3 | cytoplasmic polyadenylation element binding protein 3 | Cytoplasm | translation regulator |
| CRADD | CASP2 and RIPK1 domain containing adaptor with death domain | Cytoplasm | other |
| CXCL8 | chemokine (C-X-C motif) ligand 8 | Extracellular Space | cytokine |
| DDX17 | DEAD (Asp-Glu-Ala-Asp) box helicase 17 | Nucleus | enzyme |
| DESI1 | desumoylating isopeptidase 1 | Other | other |
| DIO1 | deiodinase, iodothyronine, type I | Cytoplasm | enzyme |
| DNASE1L1 | deoxyribonuclease I-like 1 | Cytoplasm | enzyme |
| DSC2 | desmocollin 2 | Plasma Membrane | other |
| EML5 | echinoderm microtubule associated protein like 5 | Cytoplasm | other |
| EPHA6 | EPH receptor A6 | Plasma Membrane | kinase |
| ETF1 | eukaryotic translation termination factor 1 | Cytoplasm | translation regulator |
| ETNK1 | ethanolamine kinase 1 | Cytoplasm | kinase |
| FAM154A | family with sequence similarity 154, member A | Other | other |
| FAM207A | family with sequence similarity 207, member A | Other | other |
| FAM221A | family with sequence similarity 221, member A | Other | other |
| FBF1 | Fas (TNFRSF6) binding factor 1 | Nucleus | other |
| FBXO38 | F-box protein 38 | Other | other |
| FEZ1 | fasciculation and elongation protein zeta 1 (zygin I) | Cytoplasm | other |
| FKBP1A | FK506 binding protein 1A, 12kDa | Cytoplasm | enzyme |
| FMNL2 | formin-like 2 | Cytoplasm | other |
| FOXR1 | forkhead box R1 | Nucleus | transcription regulator |
| GAS2L2 | growth arrest-specific 2 like 2 | Cytoplasm | other |
| GET4 | golgi to ER traffic protein 4 homolog (S. cerevisiae) | Cytoplasm | other |
| GLRX2 | glutaredoxin 2 | Cytoplasm | enzyme |
| GLTSCR1L | GLTSCR1-like | Other | other |
| GNAO1 | guanine nucleotide binding protein (G protein), alpha activating activity polypeptide O | Plasma Membrane | enzyme |
| GNAT1 | guanine nucleotide binding protein (G protein), alpha transducing activity polypeptide 1 | Plasma Membrane | enzyme |
| GPR50 | G protein-coupled receptor 50 | Plasma Membrane | G-protein coupled receptor |
| GRM6 | glutamate receptor, metabotropic 6 | Plasma Membrane | G-protein coupled receptor |
| HIGD2B | HIG1 hypoxia inducible domain family, member 2B | Other | other |
| HLF | hepatic leukemia factor | Nucleus | transcription regulator |
| HN1 | hematological and neurological expressed 1 | Nucleus | other |
| HNRNPA3P1 | heterogeneous nuclear ribonucleoprotein A3 pseudogene 1 | Nucleus | other |
| HSP90AB4P | heat shock protein 90kDa alpha (cytosolic), class B member 4, pseudogene | Other | other |
| IGFL1 | IGF-like family member 1 | Extracellular Space | other |
| IGSF11 | immunoglobulin superfamily, member 11 | Plasma Membrane | other |
| INO80C | INO80 complex subunit C | Nucleus | other |
| JUP | junction plakoglobin | Plasma Membrane | other |
| KIF18A | kinesin family member 18A | Cytoplasm | enzyme |
| KIF3B | kinesin family member 3B | Cytoplasm | transporter |
| KIR3DL3 | killer cell immunoglobulin-like receptor, three domains, long cytoplasmic tail, 3 | Other | other |
| KPNA2 | karyopherin alpha 2 (RAG cohort 1, importin alpha 1) | Nucleus | transporter |
| LACTB | lactamase, beta | Cytoplasm | other |
| LHX5 | LIM homeobox 5 | Nucleus | transcription regulator |
| LOC648691 | uncharacterized LOC648691 | Other | other |
| LSM12 | LSM12 homolog (S. cerevisiae) | Other | other |
| MAPK6 | mitogen-activated protein kinase 6 | Cytoplasm | kinase |
| MASP1 | mannan-binding lectin serine peptidase 1 (C4/C2 activating component of Ra-reactive factor) | Extracellular Space | peptidase |
| MED14 | mediator complex subunit 14 | Nucleus | transcription regulator |
| MTFMT | mitochondrial methionyl-tRNA formyltransferase | Cytoplasm | enzyme |
| NRXN1 | neurexin 1 | Plasma Membrane | transporter |
| NSD1 | nuclear receptor binding SET domain protein 1 | Nucleus | transcription regulator |
| OR2T2/OR2T35 | olfactory receptor, family 2, subfamily T, member 35 | Plasma Membrane | G-protein coupled receptor |
| OR2T27 | olfactory receptor, family 2, subfamily T, member 27 | Plasma Membrane | other |
| OR2W3 | olfactory receptor, family 2, subfamily W, member 3 | Plasma Membrane | G-protein coupled receptor |
| OR5W2 | olfactory receptor, family 5, subfamily W, member 2 | Plasma Membrane | G-protein coupled receptor |
| PCDHGA9 | protocadherin gamma subfamily A, 9 | Other | other |
| PER3 | period circadian clock 3 | Nucleus | other |
| PILRB | paired immunoglobin-like type 2 receptor beta | Plasma Membrane | other |
| POLR1C | polymerase (RNA) I polypeptide C, 30kDa | Nucleus | enzyme |
| PPP2R2C | protein phosphatase 2, regulatory subunit B, gamma | Other | phosphatase |
| PPP2R5D | protein phosphatase 2, regulatory subunit B', delta | Nucleus | phosphatase |
| PREP | prolyl endopeptidase | Cytoplasm | peptidase |
| PWWP2B | PWWP domain containing 2B | Other | other |
| RAB37 | RAB37, member RAS oncogene family | Cytoplasm | enzyme |
| RAB43 | RAB43, member RAS oncogene family | Cytoplasm | enzyme |
| RABL2B | RAB, member of RAS oncogene family-like 2B | Other | enzyme |
| RALGAPB | Ral GTPase activating protein, beta subunit (non-catalytic) | Other | other |
| RFESD | Rieske (Fe-S) domain containing | Other | other |
| RGPD4 (includes others) | RANBP2-like and GRIP domain containing 5 | Nucleus | other |
| RND3 | Rho family GTPase 3 | Cytoplasm | enzyme |
| RNF126P1 | ring finger protein 126 pseudogene 1 | Other | other |
| RNF17 | ring finger protein 17 | Cytoplasm | other |
| RPL8 | ribosomal protein L8 | Other | other |
| RUFY4 | RUN and FYVE domain containing 4 | Other | other |
| SBK1 | SH3 domain binding kinase 1 | Other | kinase |
| SCARA3 | scavenger receptor class A, member 3 | Plasma Membrane | transmembrane receptor |
| SECISBP2 | SECIS binding protein 2 | Cytoplasm | translation regulator |
| SELK | selenoprotein K | Cytoplasm | other |
| SERPINB8 | serpin peptidase inhibitor, clade B (ovalbumin), member 8 | Cytoplasm | other |
| SHC1 | SHC (Src homology 2 domain containing) transforming protein 1 | Cytoplasm | kinase |
| SIRT7 | sirtuin 7 | Nucleus | enzyme |
| SLC39A3 | solute carrier family 39 (zinc transporter), member 3 | Plasma Membrane | transporter |
| SLC45A1 | solute carrier family 45, member 1 | Other | transporter |
| SMDT1 | single-pass membrane protein with aspartate-rich tail 1 | Extracellular Space | other |
| SMEK3P | SMEK homolog 3, suppressor of mek1 (Dictyostelium) pseudogene | Other | other |
| SNAPC4 | small nuclear RNA activating complex, polypeptide 4, 190kDa | Nucleus | transcription regulator |
| SNORD31 | small nucleolar RNA, C/D box 31 | Other | other |
| SNX12 | sorting nexin 12 | Cytoplasm | transporter |
| SOCS2 | suppressor of cytokine signaling 2 | Cytoplasm | other |
| SPATA18 | spermatogenesis associated 18 | Cytoplasm | other |
| SPHKAP | SPHK1 interactor, AKAP domain containing | Cytoplasm | other |
| SSSCA1 | Sjogren syndrome/scleroderma autoantigen 1 | Other | other |
| STX3 | syntaxin 3 | Plasma Membrane | transporter |
| SUN2 | Sad1 and UNC84 domain containing 2 | Nucleus | other |
| TAC1 | tachykinin, precursor 1 | Extracellular Space | other |
| TBC1D3 (includes others) | TBC1 domain family, member 3H | Extracellular Space | other |
| TDGF1 | teratocarcinoma-derived growth factor 1 | Extracellular Space | growth factor |
| TFAP2A | transcription factor AP-2 alpha (activating enhancer binding protein 2 alpha) | Nucleus | transcription regulator |
| TNFAIP1 | tumor necrosis factor, alpha-induced protein 1 (endothelial) | Plasma Membrane | ion channel |
| TNRC6B | trinucleotide repeat containing 6B | Other | other |
| TP53I3 | tumor protein p53 inducible protein 3 | Cytoplasm | enzyme |
| TRABD | TraB domain containing | Extracellular Space | other |
| TRAF5 | TNF receptor-associated factor 5 | Cytoplasm | transporter |
| TRIM37 | tripartite motif containing 37 | Cytoplasm | enzyme |
| TUBA1A | tubulin, alpha 1a | Cytoplasm | other |
| VMP1 | vacuole membrane protein 1 | Plasma Membrane | other |
| XPO5 | exportin 5 | Nucleus | transporter |
| ZDHHC22 | zinc finger, DHHC-type containing 22 | Other | other |
| ZNF862 | zinc finger protein 862 | Other | other |
| ZPBP2 | zona pellucida binding protein 2 | Extracellular Space | other |
| ZSWIM6 | zinc finger, SWIM-type containing 6 | Other | other |
