# Supplementary material for: Optimal targeting of BCL-family proteins in head and neck squamous cell carcinoma requires inhibition of both BCL-xL and MCL-1
Source: Oncotarget. 2019 Jan 11;10(4):494–510. doi: 10.18632/oncotarget.26563 (PMC6355180; doi:10.18632/oncotarget.26563)
Supplement: Supplementary file 3 [file oncotarget-10-494-s003.docx]

**Supplementary Table 2: Canonical pathways identified using Ingenuity Pathway analysis to evaluate 135 genes differentiating treatment-resistant from treatment-sensitive head and neck squamous cell carcinoma**

| ***Ingenuity Canonical Pathways*** | ***-log***  ***(p-value)*** | ***Ratio*** | ***Molecules*** | |  |  |  |  |  |  |
| --- | --- | --- | --- | --- | --- | --- | --- | --- | --- | --- |
| PI3K/AKT Signaling | 3.06E00 | 4.07E-02 | SHC1,BCL2L1,PPP2R5D,CDKN1A,PPP2R2C | | | | | |  | * |
| JAK/Stat Signaling | 3.04E00 | 5.56E-02 | SHC1,BCL2L1,CDKN1A,SOCS2 | | | |  |  |  | * |
| Cyclins and Cell Cycle Regulation | 2.91E00 | 5.13E-02 | PPP2R5D,CDKN1A,PPP2R2C,CDKN2B | | | | |  |  |  |
| Molecular Mechanisms of Cancer | 2.8E00 | 2.19E-02 | SHC1,BCL2L1,CASP3,GNAT1,RND3,GNAO1,CDKN1A,CDKN2B | | | | | | | * |
| Synaptic Long Term Depression | 2.8E00 | 3.55E-02 | GNAT1,PPP2R5D,GNAO1,GRM6,PPP2R2C | | | | | |  |  |
| Telomerase Signaling | 2.53E00 | 4.04E-02 | SHC1,PPP2R5D,CDKN1A,PPP2R2C | | | | |  |  |  |
| Endothelin-1 Signaling | 2.44E00 | 2.92E-02 | SHC1,CASP3,GNAT1,GNAO1,MAPK6 | | | | |  |  |  |
| CD27 Signaling in Lymphocytes | 2.43E00 | 5.77E-02 | BCL2L1,CASP3,TRAF5 | | |  |  |  |  | * |
| Lymphotoxin β Receptor Signaling | 2.38E00 | 5.56E-02 | BCL2L1,CASP3,TRAF5 | | |  |  |  |  | * |
| Role of CHK Proteins in Cell Cycle Checkpoint Control | 2.36E00 | 5.45E-02 | PPP2R5D,CDKN1A,PPP2R2C | | | |  |  |  |  |
| PTEN Signaling | 2.26E00 | 3.39E-02 | SHC1,BCL2L1,CASP3,CDKN1A | | | |  |  |  | * |
| Breast Cancer Regulation by Stathmin1 | 2.24E00 | 2.62E-02 | SHC1,TUBA1A,PPP2R5D,CDKN1A,PPP2R2C | | | | | |  |  |
| IL-15 Signaling | 2.14E00 | 4.55E-02 | CXCL8,SHC1,BCL2L1 | | |  |  |  |  | * |
| Small Cell Lung Cancer Signaling | 2.05E00 | 4.23E-02 | BCL2L1,TRAF5,CDKN2B | | |  |  |  |  | * |
| Lipid Antigen Presentation by CD1 | 1.98E00 | 7.69E-02 | CANX,AP2S1 | |  |  |  |  |  |  |
| CTLA4 Signaling in Cytotoxic T Lymphocytes | 1.8E00 | 3.41E-02 | PPP2R5D,PPP2R2C,AP2S1 | | | |  |  |  |  |
| G Beta Gamma Signaling | 1.8E00 | 3.41E-02 | SHC1,GNAT1,GNAO1 | | |  |  |  |  |  |
| Thyronamine and Iodothyronamine Metabolism | 1.75E00 | 3.33E-01 | DIO1 |  |  |  |  |  |  |  |
| Thyroid Hormone Metabolism I (via Deiodination) | 1.75E00 | 3.33E-01 | DIO1 |  |  |  |  |  |  |  |
| Cell Cycle Regulation by BTG Family Proteins | 1.73E00 | 5.71E-02 | PPP2R5D,PPP2R2C | | |  |  |  |  |  |
| Role of JAK2 in Hormone-like Cytokine Signaling | 1.73E00 | 5.71E-02 | SHC1,SOCS2 | |  |  |  |  |  |  |
| Glioma Signaling | 1.71E00 | 3.16E-02 | SHC1,CDKN1A,CDKN2B | | |  |  |  |  |  |
| CREB Signaling in Neurons | 1.71E00 | 2.34E-02 | SHC1,GNAT1,GNAO1,GRM6 | | | |  |  |  |  |
| Glucocorticoid Receptor Signaling | 1.69E00 | 1.92E-02 | CXCL8,SHC1,BCL2L1,CDKN1A,MED14 | | | | |  |  | * |
| Ephrin Receptor Signaling | 1.69E00 | 2.3E-02 | EPHA6,SHC1,GNAT1,GNAO1 | | | |  |  |  |  |
| p53 Signaling | 1.68E00 | 3.06E-02 | BCL2L1,CDKN1A,TP53I3 | | |  |  |  |  |  |
| CDK5 Signaling | 1.67E00 | 3.03E-02 | PPP2R5D,MAPK6,PPP2R2C | | | |  |  |  |  |
| Docosahexaenoic Acid (DHA) Signaling | 1.64E00 | 5.13E-02 | BCL2L1,CASP3 | |  |  |  |  |  | * |
| Arsenate Detoxification I (Glutaredoxin) | 1.63E00 | 2.5E-01 | GLRX2 |  |  |  |  |  |  |  |
| Biotin-carboxyl Carrier Protein Assembly | 1.63E00 | 2.5E-01 | ACACB |  |  |  |  |  |  |  |
| Role of PKR in Interferon Induction and Antiviral Response | 1.62E00 | 5E-02 | CASP3,TRAF5 | |  |  |  |  |  |  |
| ILK Signaling | 1.59E00 | 2.15E-02 | CASP3,RND3,PPP2R5D,PPP2R2C | | | | |  |  |  |
| Pancreatic Adenocarcinoma Signaling | 1.59E00 | 2.83E-02 | BCL2L1,CDKN1A,CDKN2B | | | |  |  |  | * |
| mTOR Signaling | 1.58E00 | 2.13E-02 | RND3,PPP2R5D,FKBP1A,PPP2R2C | | | | |  |  |  |
| Thrombin Signaling | 1.56E00 | 2.09E-02 | SHC1,GNAT1,RND3,GNAO1 | | | |  |  |  |  |
| Role of Tissue Factor in Cancer | 1.55E00 | 2.73E-02 | CXCL8,BCL2L1,CASP3 | | |  |  |  |  | * |
| Androgen Signaling | 1.54E00 | 2.7E-02 | SHC1,GNAT1,GNAO1 | | |  |  |  |  |  |
| p70S6K Signaling | 1.46E00 | 2.52E-02 | SHC1,PPP2R5D,PPP2R2C | | | |  |  |  |  |
| TNFR1 Signaling | 1.46E00 | 4.08E-02 | CASP3,CRADD | |  |  |  |  |  |  |
| AMPK Signaling | 1.33E00 | 2.24E-02 | ACACB,PPP2R5D,PPP2R2C | | | |  |  |  |  |
| Airway Pathology in Chronic Obstructive Pulmonary Disease | 1.33E00 | 1.25E-01 | CXCL8 |  |  |  |  |  |  |  |
| Myc Mediated Apoptosis Signaling | 1.33E00 | 3.45E-02 | SHC1,CASP3 | |  |  |  |  |  |  |
| Induction of Apoptosis by HIV1 | 1.3E00 | 3.33E-02 | BCL2L1,CASP3 | |  |  |  |  |  |  |
| Assembly of RNA Polymerase I Complex | 1.28E00 | 1.11E-01 | POLR1C | |  |  |  |  |  |  |
| Phosphatidylethanolamine Biosynthesis II | 1.28E00 | 1.11E-01 | ETNK1 |  |  |  |  |  |  |  |
| GM-CSF Signaling | 1.28E00 | 3.23E-02 | SHC1,BCL2L1 | |  |  |  |  |  | * |
| Cell Cycle: G1/S Checkpoint Regulation | 1.25E00 | 3.12E-02 | CDKN1A,CDKN2B | | |  |  |  |  |  |
| Regulation of eIF4 and p70S6K Signaling | 1.24E00 | 2.05E-02 | SHC1,PPP2R5D,PPP2R2C | | | |  |  |  |  |
| Glioblastoma Multiforme Signaling | 1.24E00 | 2.05E-02 | SHC1,RND3,CDKN1A | | |  |  |  |  |  |
| Mitotic Roles of Polo-Like Kinase | 1.23E00 | 3.03E-02 | PPP2R5D,PPP2R2C | | |  |  |  |  |  |
| CXCR4 Signaling | 1.2E00 | 1.97E-02 | GNAT1,RND3,GNAO1 | | |  |  |  |  |  |
| Tec Kinase Signaling | 1.16E00 | 1.9E-02 | GNAT1,RND3,GNAO1 | | |  |  |  |  |  |
| Prolactin Signaling | 1.15E00 | 2.74E-02 | SHC1,SOCS2 | |  |  |  |  |  |  |
| Ephrin B Signaling | 1.15E00 | 2.74E-02 | GNAT1,GNAO1 | |  |  |  |  |  |  |
| STAT3 Pathway | 1.15E00 | 2.74E-02 | CDKN1A,SOCS2 | |  |  |  |  |  |  |
| Germ Cell-Sertoli Cell Junction Signaling | 1.15E00 | 1.88E-02 | TUBA1A,RND3,JUP | | |  |  |  |  |  |
| Role of IL-17A in Psoriasis | 1.13E00 | 7.69E-02 | CXCL8 |  |  |  |  |  |  |  |
| PDGF Signaling | 1.11E00 | 2.6E-02 | SHC1,ABL2 | |  |  |  |  |  |  |
| Dopamine Receptor Signaling | 1.1E00 | 2.56E-02 | PPP2R5D,PPP2R2C | | |  |  |  |  |  |
| Wnt/β-catenin Signaling | 1.09E00 | 1.78E-02 | PPP2R5D,GNAO1,PPP2R2C | | | |  |  |  |  |
| Ceramide Signaling | 1.08E00 | 2.5E-02 | PPP2R5D,PPP2R2C | | |  |  |  |  |  |
| RhoGDI Signaling | 1.07E00 | 1.73E-02 | GNAT1,RND3,GNAO1 | | |  |  |  |  |  |
| Granzyme B Signaling | 1.04E00 | 6.25E-02 | CASP3 |  |  |  |  |  |  |  |
| Parkinson's Signaling | 1.04E00 | 6.25E-02 | CASP3 |  |  |  |  |  |  |  |
| Production of Nitric Oxide and Reactive Oxygen Species in Macrophages | 1.03E00 | 1.67E-02 | RND3,PPP2R5D,PPP2R2C | | | |  |  |  |  |
| HIPPO signaling | 1.03E00 | 2.33E-02 | PPP2R5D,PPP2R2C | | |  |  |  |  |  |
| Bladder Cancer Signaling | 1.02E00 | 2.3E-02 | CXCL8,CDKN1A | |  |  |  |  |  |  |
| IL-8 Signaling | 1.01E00 | 1.64E-02 | CXCL8,BCL2L1,RND3 | | |  |  |  |  | * |
| RAN Signaling | 1.01E00 | 5.88E-02 | KPNA2 |  |  |  |  |  |  |  |
| UVA-Induced MAPK Signaling | 1.01E00 | 2.27E-02 | BCL2L1,CASP3 | |  |  |  |  |  | * |
| PAK Signaling | 1E00 | 2.25E-02 | SHC1,CASP3 | |  |  |  |  |  |  |
| OX40 Signaling Pathway | 1E00 | 2.25E-02 | BCL2L1,TRAF5 | |  |  |  |  |  | * |
| Apoptosis Signaling | 1E00 | 2.25E-02 | BCL2L1,CASP3 | |  |  |  |  |  | * |

*17 pathways included BCL-xL (BCL2L1).
